# Prevalence of non-febrile seizures in children with idiopathic autism spectrum disorder and their unaffected siblings: a retrospective cohort study

**DOI:** 10.1186/s12883-016-0764-3

**Published:** 2016-11-28

**Authors:** Lena M. McCue, Louise H. Flick, Kimberly A. Twyman, Hong Xian, Thomas E. Conturo

**Affiliations:** 1 Division of Biostatistics, Washington University in St. Louis, School of Medicine, 660 Euclid Ave., St. Louis, MO 63110 USA; 2 Epidemiology Department, Saint Louis University, College for Public Health and Social Justice, 3545 Lafayette Ave., St. Louis, MO 63104 USA; 3 Department of Pediatrics, Saint Louis University School of Medicine, 1465 S Grand Blvd., St Louis, MO 63104 USA; 4 Department of Biostatistics, Saint Louis University, College for Public Health and Social Justice, 3545 Lafayette Ave., St. Louis, MO 63104 USA; 5 Department of Radiology, Washington University School of Medicine, 4525 Scott Ave., St Louis, MO 63110 USA

**Keywords:** Autism, Idiopathic, Seizures, Epilepsy, Genetic, Prevalence, Familial, Siblings, AGRE

## Abstract

**Background:**

Autism spectrum disorder (ASD) is a heterogeneous disorder characterized not only by deficits in communication and social interactions but also a high rate of co-occurring disorders, including metabolic abnormalities, gastrointestinal and sleep disorders, and seizures. Seizures, when present, interfere with cognitive development and are associated with a higher mortality rate in the ASD population.

**Methods:**

To determine the relative prevalence of non-febrile seizures in children with idiopathic ASD from multiplex and simplex families compared with the unaffected siblings in a cohort of 610 children with idiopathic ASD and their 160 unaffected siblings, participating in the Autism Genetic Resource Exchange project, the secondary analysis was performed comparing the life-time prevalence of non-febrile seizures. Statistical models to account for non-independence of observations, inherent with the data from multiplex families, were used in assessing potential confounding effects of age, gender, and history of febrile seizures on odds of having non-febrile seizures.

**Results:**

The life-time prevalence of non-febrile seizures was 8.2% among children with ASD and 2.5% among their unaffected siblings. In a logistic regression analysis that adjusted for familial clustering, children with ASD had 5.27 (95%CI: 1.51–18.35) times higher odds of having non-febrile seizures compared to their unaffected siblings. In this comparison, age, presence of gastrointestinal dysfunction, and history of febrile seizures were significantly associated with the prevalence of non-febrile seizures.

**Conclusion:**

Children with idiopathic ASD are significantly more likely to have non-febrile seizures than their unaffected siblings, suggesting that non-febrile seizures may be ASD-specific. Further studies are needed to determine modifiable risk factors for non-febrile seizures in ASD.

## Background

Autism spectrum disorder (ASD) is a neurodevelopmental disorder characterized not only by the deficits in communication and social interaction but also by the high prevalence of co-occurring medical conditions such as seizures and epilepsy, metabolic abnormalities, gastrointestinal dysfunctions, and sleep disorders [1–6]. Epilepsy is a serious chronic condition often co-occurring with ASD. When present, it interferes with motor and cognitive development, is linked to poor long-term health outcomes, and is associated with higher mortality rates [7, 8]. Costs of care and education for individuals with ASD are even higher when epilepsy is present [9–11].

The estimates of seizures and epilepsy rates in children with ASD range from 7% (Interactive Autism Network registry) to 15% (population-based Autism and Developmental Disabilities Monitoring Network) [12, 13]. Rates of seizures for clinical ASD populations have been reported as high as 38% [8, 14, 15]. The wide range in reported prevalence may be attributed to differences between the studies samples with respect to age, definition of seizures, referral bias (common in studies with clinical populations), inclusion of individuals with non-idiopathic ASD diagnoses, as well as methodological differences across the studies. Prior studies of seizures in children with ASD also did not separate febrile seizures from non-febrile. Nevertheless, even by the most conservative estimates, these rates are higher than those in the general population [16]. The relationship between epilepsy and ASD continues to be a controversial topic, and is clinically important for a number of reasons, including the fact that seizures in ASD tend to be refractory to treatment [17]. The risk of developing seizures increases with age in ASD, whereas this risk decreases with age in other neurodevelopmental disorders that are associated with high rates of epilepsy (e.g., cerebral palsy) [14, 17]. The unusual age and treatment response suggest that risk factors for seizures in ASD differ from risk factors in other developmental disorders.

To bridge the gap in current knowledge, this study assesses the relative prevalence of non-febrile seizures in a large cohort of children age 2 to 18 years with well-documented idiopathic ASD compared with their unaffected siblings from the Autism Genetic Resource Exchange (AGRE). We hypothesize that children with idiopathic ASD will be more likely to have reported at least one non-febrile seizure episode compared to their unaffected siblings.

Despite the growing body of scientific literature on prevalence of non-febrile seizures in children with ASD, to date few studies assessed febrile and non-febrile seizures separately. Yet such distinction is critical since the pathophysiology and risk factors associated with febrile seizures differ from other types of seizures, and febrile seizures are known to increase the risk of developing non-febrile seizures later in life [18–20]. Also, when assessing seizure prevalence, it is important to distinguish whether the ASD diagnosis is primary (idiopathic) or secondary to another neurological disorder (e.g. cerebral palsy, tuberous sclerosis, or fragile X syndrome), as the seizure etiology may differ from idiopathic ASD in secondary cases. Further, in order to determine whether the observed prevalence of seizures is specific to ASD, use of an appropriate comparison group is imperative. Employing a general population as a reference is likely to overestimate the prevalence of seizures in the ASD population because differences arising from a shared environment and shared genetic characteristics are not accounted for in such a comparison. Since siblings share, on average, fifty percent of genes as well as the same environment, the use of typically-developing siblings as a reference group allows for control of these factors. This approach will produce more conservative and accurate prevalence estimates, while enabling the distinction of phenotypical characteristics due to familial aggregation from those specific to ASD.

## Methods

### Study design

We conducted a secondary analysis of data from a registry-based retrospective cohort study of 731 children with ASD and their 192 children unaffected siblings from the AGRE project for whom phenotypic data were collected [21]. Permission to download data was granted by AGRE. These data were collected for the AGRE study from patient records, parental reports, and on-site home visits. AGRE is one of the largest DNA repositories and family registries of ASD, housing a database of genotypic and phenotypic information. AGRE houses a collection from over 1700 well-characterized families with one or more children with ASD. During recruitment, families identified the diagnosis they received from their health care professional or autism specialist. Families with one or more children with autism, pervasive developmental disorder not otherwise specified or Asperger’s syndrome diagnosed under DSM-IV-TR were accepted into the program, provided they had not contributed to any other autism gene bank. This study estimated the prevalence of non-febrile seizures in children with idiopathic ASD compared to the unaffected siblings as of the last date of AGRE data included in the dataset.

### Sample

The study sample was drawn from the subset of AGRE participants registered with AGRE from 1999 to 2009 (year AGRE suspended further recruitment), and for whom phenotypic data were collected. This population includes children diagnosed with an ASD from families with one or more affected children and their linked unaffected siblings, for whom medical histories, environmental exposure histories, and physical/neurological exam data were available. Data that flagged possible non-idiopathic autism were also collected by AGRE using the following indicators: known neurogenic disorders, abnormal neurological exams, abnormal molecular or medical tests, focal abnormality on structural imaging, significant dysmorphology, known chromosomal abnormality, fragile X (full mutation), pre- or peri-natal injuries, prematurity less than 35 weeks, or small nuclear ribonucleoprotein polypeptide N (SNRPN) duplications.

### Inclusion and exclusion criteria

Children were included in the study sample if they were age 2 to 18 years and had a confirmed ASD diagnosis. A minimum age of 2 was chosen to ensure that diagnostic criteria involving language function could be evaluated accurately. Also included were their unaffected siblings, age 2 to 18 years, registered within the same period. Not every child with ASD had an unaffected sibling.

Children with diagnoses of tuberous sclerosis, Landau-Kleffner Syndrome, Rett syndrome, cerebral palsy, Lennox-Gastaut syndrome, West syndrome, and continuous spike-and-wave syndrome (CSWS) were excluded as having conditions indicative of secondary ASD, as well as independent risk factors for epilepsy. Individuals with history of status epilepticus [22], neonatal seizures, infantile spasms, and neurofibromatosis-1 (NF-1) were also excluded because such history may be indicative of severe brain abnormalities and/or secondary autism. Subjects flagged by AGRE as having possible non-idiopathic autism were also excluded.

### Measures

The ASD diagnosis received by participants from a physician was verified by AGRE using the Autism Diagnostic Observation Schedule (ADOS) and the Autism Diagnostic Interview-revised (ADI-R), both based on DSM-IV diagnostic criteria [23–26]. AGRE data also includes ADOS/ADI-R test scores for the unaffected siblings. In our sample, everyone in the ASD-affected group had a diagnosis of an ASD prior to enrolment with AGRE and had met the following criteria during AGRE assessment: (i) cutoff points for autism on ADI-R and/or ADOS; or (ii) cutoff points for autism spectrum on ADOS. None of the unaffected siblings in our sample had met the ASD cutoff points on ADOS or ADI-R.

#### Autism Diagnostic Observation Schedule (ADOS)

The ADOS is a semi-structured assessment of communication, social interaction, repetitive behavior, and play characteristics for individuals suspected of having autism or other pervasive developmental disorders. The ADOS is based on the examiner’s observation of the individual, and consists of four modules, each of which is appropriate for children and adults of differing developmental and language levels, ranging from nonverbal to verbally-fluent [23].

#### Autism Diagnostic Interview-Revised (ADI-R)

The ADI-R is a clinical diagnostic instrument for assessing autism in children and adults. The ADI-R is based on historical data from parent interviews, and provides a diagnostic algorithm for autism as described in both the ICD-10 and DSM-IV. The instrument focuses on behavior in three main areas: qualities of reciprocal social interaction; communication and language; and restricted and repetitive, stereotyped interests and behaviors. The ADI-R is appropriate for children and adults with mental ages from about 18 months and above [27]. Both, ADOS and ADI-R were administered by AGRE physicians, trained by a research-certified ADOS/ADI-R trainer, who underwent reliability reviews throughout the data collection process.

Lastly, AGRE pediatric neurologists conducted an in-home structured medical history interview to collect retrospective health information on each study participant. More detailed medical information was collected for the affected children than unaffected. The interview questions relevant to this study are included in Table 1.

**Table 1:** Medical History Questions

For ASD Affected Child	For Unaffected Sibling
Neurological – Is there any known issue/abnormality in this area?	Neurological – Is there any known issue/abnormality in this area?
1. Febrile seizures – “yes”, “no”?	1. --------------------------------
2. Other seizures – “yes”, “no”?	2. Seizures – “yes”, “no”?
3. Seizures type: complex partial, febrile, generalized/GTC/grand mal, absence/petit/mal, infantile spasms, other, unknown, multiple?	3. Seizures type: complex partial, febrile, generalized/GTC/grand mal, absence/petit/mal, infantile spasms, other, unknown, multiple?
4. Age of seizure onset (years)?	4. Age of seizure onset (years)?
5. Number of seizures?	5. ----------------------------------
6. Frequency of seizures?	6. ----------------------------------
7. Seizures requiring treatment with medication?	7. ----------------------------------
8. Cerebral abnormalities – “yes”, “no”?	8. ----------------------------------
9. Comments about cerebral abnormalities.	9. ----------------------------------

In our study, the outcome variable was coded as “yes” if the AGRE records indicated presence of at least one non-febrile seizure that was not classified as infantile spasms. That is, children were not excluded from the study sample if they had a history of febrile seizures, but to be included in the seizure positive group for testing the study hypothesis, they had to have had at least one seizure episode not due to fever. To produce a conservative estimate of prevalence, those who did not report having seizures or who provided no response on the seizure type variable were coded as negative on our outcome variable. This rationale was based on two assumptions: (a) those who had seizures would have reported them; and (b) those who had no seizures would not have necessarily responded to the question. Presence of febrile seizures was defined as at least one seizure episode provoked by fever reported in AGRE records. Gastrointestinal (GI) dysfunctions were defined as presence of at least one GI disorder symptom in AGRE records.

### Statistical analyses

All analyses were performed using SAS version 9.4. Frequency distributions and prevalence rates were calculated for each of the characteristics of interest. The AGRE data includes families with more than one child with ASD so not all observations were independent. The non-independence of observations was accounted for in all analyses by adjusting variance estimations using SAS PROC SURVEY procedures (PROC SURVEYFREQ and PROC SURVEYLOGISTIC). The SAS PROC SURVEY methods were developed for complex survey data in which the assumptions of random population sampling and independence of observations are not met. To measure the association between non-febrile seizures and ASD affected status logistic regression analysis was used, treating each family as a cluster. This approach accounted for random effects of the within-family covariates and, subsequently, adjusted the variance, significance levels, and confidence intervals for family clusters. It also allowed full statistical control for the main effects of measured and unmeasured familial confounders.

Confounding effects of febrile seizures and GI dysfunctions were statistically controlled for in our analyses. Bivariate analyses were performed to measure the association of non-febrile seizures with affected or unaffected ASD status. A prevalence odds ratio was calculated to test for a significant difference. Categorical variables were assessed using Rao-Scott adjusted χ^2^ test for association. Group means were assessed using cluster-adjusted Wald F tests for linear regression models. All statistical tests were 2-tailed with statistical significance attained when the 2-tailed *p*-value was less than .05, unless otherwise stated.

## Results

### Sample characteristics

After selection criteria were applied, the total sample comprised 770 children age 2 to 18 years with a mean age of 8.48 years (SE = 0.16) (Table 2). The children were from 324 families with each family contributing at least one child with ASD: 72 families (22.2%, 72/324) had one child with ASD, 224 (69.1%, 224/324) had two children with ASD and 28 (8.6%, 28/324) families had 3 or more children with ASD. Not all of the 324 families included an unaffected sibling, 197 families (60.8%, 197/324) included only a child or children with ASD and no unaffected siblings, 101 included one unaffected sibling (31.2%, 101/324), 21 included two unaffected siblings (6.5%, 21/324) and 5 families included 3 or more unaffected siblings (1.5%, 5/324).

**Table 2:** Sample Characteristics by ASD Affected or Unaffected status (*N* = 770)

Characteristic variable	ASD (n = 610) # (%)	Unaffected siblings (n = 160) # (%)	F or χ^2^; *p*
Gender			
Male	477 (78.1)	73 (44.2)	χ^2^_(1)_ = 61.08; <0.0001
Female	134 (21.9)	92 (55.8)	
Mean age (yr.) at AGRE medical assessment	8.34 (SE 0.16)	9.01 (SE 0.35)	F_(1)_ = 1.35; 0.25
^a^Race			
White	487 (79.7)	135 (81.8)	χ^2^_(2)_ = 1.11; 0.57
Asian	26 (4.3)	8 (4.9)	
Other race, multiple race, or unknown	98 (16.0)	22 (13.3)	
^a^Ethnicity			
Hispanic or Latino	115 (18.8)	34 (20.6)	χ^2^_(2)_ = 0.40; 0.82
Not Hispanic or Latino	474 (77.6)	126 (76.4)	
Unknown	22 (3.6)	5 (3.0)	
Maternal education			
≤ High school	53 (9.2)	13 (8.1)	χ^2^_(2)_ = 1.03; 0.60
Some college or graduated from college	392 (68.8)	115 (71.9)	
Some grad school or completed grad school	133 (18.0)	32 (20.0)	
Paternal education			
≤ High school	75 (13.2)	27 (16.9)	χ^2^_(2)_ = 1.81; 0.40
Some college or graduated from college	332 (58.3)	93 (58.1)	
Some grad school or completed grad school	162 (28.5)	40 (25.0)	

^a^Race and Ethnicity categories were collapsed to avoid empty cells. Race categories “American Indian/Alaskan Native” *n* = 2, “Asian” *n* = 26, “Black or African American” *n* = 8, “More than One Race” *n* = 46, “Native Hawaiian or other Pacific Islander” *n* = 6, “Unknown” *n* = 36 were combined under “Other race, multiple race, or unknown”. For our Ethnicity variable, the category “Unknown” *n* = 22 was set to missing

The male to female ratio was 3.55 to1 in the ASD group compared to 1 to1 in the unaffected group (71.2% boys and 28.8% girls). This ratio is consistent with the known 3:1 to 4:1 male-female ratio for the full spectrum of ASD [28, 29]. Of the 770 participants, 79.2% (610) were children with ASD, and 20.8% (160) were their unaffected siblings. In the ASD-affected group, 582 (95.4%) children were from families with more than one family member affected with ASD (multiplex families), and 28 (4.6%) were from families with only one family member affected with ASD (simplex families). In the unaffected siblings group, 140 (87.5%) were from multiplex families, and 20 (12.5%) were from simplex families. No association was observed between race, ethnicity, maternal and paternal education level and the ASD affected status.

### Seizures

The total prevalence of seizures, including febrile, was 9.4% (72/770). The overall prevalence of non-febrile seizures was 7.0% (54/770). The prevalence of non-febrile seizures in the ASD group was 8.2% (50/610) and 2.5% in the unaffected siblings (4/160). The overall prevalence of febrile seizures was 3.1% (24/770). The prevalence of febrile seizures in the ASD group was 3.1% (19/610) and 3.1% among the unaffected siblings (5/160). Among the ASD group, 1% (6/610) reported having both febrile seizures and non-febrile seizures. The mean age-at-onset for febrile seizures in the ASD group was 1.99 (SE = 0.35) years. The mean age-at-onset for non-febrile seizures in the ASD group was 4.24 (SE = 0.55). Age-at-onset was unavailable for the unaffected siblings. Among 50 children with ASD and non-febrile seizures, 48% (24/50) reported having seizures requiring pharmacological treatment, and, among these, 16.7% (4/24) reported having seizures that were refractory to treatment.

The types of non-febrile seizures reported in the ASD group were complex partial (*n* = 9; 1.5% of all ASD children), generalized / grand mal (*n* = 16; 2.6%), absence / petit mal (*n* = 7; 1.2%), other (*n* = 6; 1.0%), unknown type (*n* = 7; 1.2%), and multiple types (*n* = 5; 1.0%). Among children with ASD, 1.5% (9/610) reported having only one seizure episode, 4.6% (28/610) reported having two or more seizure episodes, and for 2.1% (13/610) episode information was unavailable. The types of non-febrile seizures among the unaffected siblings were complex partial (*n* = 1; 0.6%), absence / petit mal (*n* = 1; 0.6%), and other (*n* = 2; 1.3%) (Table 3).

**Table 3:** Primary Exposures and Outcomes by ASD Affected or Unaffected status (*N* = 770)

Characteristic variable	ASD (*n* = 610) # (%)	Unaffected siblings (*n* = 160) # (%)	F or χ^2^; *p*
Non-febrile seizures			
Yes	50 (8.2)	4 (2.5)	χ^2^_(1)_ = 6.25; 0.01
No	134 (91.8)	156 (97.5)	
Febrile seizures			
Yes	19 (3.1)	5 (3.1)	χ^2^_(1)_ = 0.0001; 0.99
No	591 (96.9)	155 (96.9)	
Type of non-febrile seizures			
Complex partial	9 (1.5)	1 (0.6)	χ^2a^
Generalized/ Grand mal	16 (2.6)	0 (0.0)	
Absence/petit mal	7 (1.1)	1 (0.6)	
Other	6 (1.0)	2 (1.3)	
Unknown	7 (1.1)	0 (0.0)	
Multiple	5 (0.8)	0 (0.0)	
None	560 (91.8)	156 (97.5)	
GI dysfunctions			
Yes	251 (42.5)	20 (12.8)	χ^2^_(2)_ = 45.33; <0.0001
No	340 (57.5)	136 (87.2)	
Type of GI dysfunctions			
Gastroesophageal reflux	12 (2.0)	6 (3.8)	χ^2^_(6)_ = 57.37; <0.0001
Irritable bowel syndrome	2 (0.3)	2 (1.3)	
Chronic diarrhea	66 (10.8)	3 (1.9)	
Constipation	86 (14.1)	3 (1.9)	
Other	33 (5.4)	3 (1.9)	
Multiple	52 (8.5)	3 (1.9)	
None	359 (58.9)	140 (87.5)	

^a^Statistics not available due to empty cells

There were significantly more non-febrile seizures reported for children with ASD (8.2%; 50/610) than for their unaffected siblings (2.5%; 4/160) (adjusted OR 4.90; 95% CI: 1.63 – 14.73) (Table 3). In the logistic regression analysis the ASD-specific unadjusted prevalence odds ratio for having non-febrile seizures was 3.48 (95% CI: 1.23–9.87) compared to the unaffected siblings (*p* <0.05) (Table 4: Model 1). The statistical model did not include any covariates.

**Table 4:** Logistic Regression Models with Variables Associated with Reported Non-Febrile Seizures (*N* = 770)

Variable	Model 1 OR (95% CI)	Model 2 OR (95% CI)	Model 3 OR (95% CI)	Model 4 OR (95% CI)	Model 5^b^ OR (95% CI)
ASD Status					
Affected	3.48 (1.23–9.87)^a^	4.00 (1.46–11.00)^a^	4.63 (1.62–13.22)^a^	4.79 (1.47–15.63)^a^	5.27 (1.51–18.35)^a^
Unaffected	Reference	------	------	------	
Gender(fem.)	-------	1.49 (0.82–2.74)	1.59 (0.86–2.93)	1.50 (0.79–2.84)	1.51 (0.78–2.91)
Age (yr.) at AGRE asses.	-------	------	1.12 (1.04–1.22)	1.11 (1.02–1.20)	1.12 (1.03–1.21)
GI dysf.	-------	-------	-------	1.91 (1.05–3.48)	1.87 (1.03–3.41)
History of febr. seizures	-------	-------	-------	-------	5.34 (1.86–15.37)

^a^Statistics represent change in the point estimate with addition of each variable into the model (one at a time) starting with gender

^b^Final model

Prior studies have identified age and gender among the risk factors for seizures in the general as well as the ASD populations [7, 30]. In addition, studies with the general population have established history of febrile seizures as a factor leading to development of non-febrile seizures later in life [18, 19]. Lastly, a study by Wang et al. (2011) found higher prevalence of GI dysfunctions in children with ASD compared to their ASD unaffected siblings, indicating the importance of controlling for GI status when assessing relative prevalence of seizures in ASD [6]. These four variables (age, gender, GI dysfunction, and history of febrile seizures) were added in the subsequent logistic regression to account for their effects on the odds of having non-febrile seizures (Table 4: Models 2, 3, 4 & 5). Age, GI dysfunctions, and history of febrile seizures were significantly associated with the outcome. Specifically, the odds of having non-febrile seizures increased with age ((OR_(age)_: 1.13; 95% CI: 1.04 – 1.23), presence of GI dysfunction (OR_(GI)_: 1.91; 95% CI: 1.05–3.48), and those with a history of febrile seizures had five times the odds of reporting non-febrile seizures OR_(febrile)_: 5.46; 95% CI: 1.96 – 15.23) (Table 3: Models 3 & 4). Gender, however, showed no significant association with the outcome in any of our models (OR_(gender)_: 1.58; 95% CI: 0.84 – 2.98). The inclusion of history of febrile seizures, age, and gender each changed the adjusted odds ratio by more than 10% and, therefore, these variables were retained in the final model [31]. In a logistic regression analysis that controlled for history of febrile seizures, age, presence of GI dysfunctions, and gender, the adjusted odds of having non-febrile seizures were 5.27 (95%CI: 1.51 – 18.53) for children with idiopathic ASD compared to their unaffected siblings (*p* < 0.01). Accordingly, children affected with ASD had five-times the odds of having non-febrile seizures compared to their unaffected siblings (Table 3).

Since our sample largely came from multiplex families (95.4% in the ASD group, and 86.1% in the unaffected siblings group) we conducted secondary analyses excluding children from simplex families in order to test whether results for multiplex families differ from the combined results. These results were consistent with those in primary analyses.

## Discussion

Our study is one of the largest studies to date estimating the prevalence of non-febrile seizures in a well-defined population of children with idiopathic ASD while controlling for familial aggregation, age, history of febrile seizures and GI dysfunctions. Our study is also one of the largest studies comparing the lifetime prevalence of non-febrile seizures between ASD affected and unaffected siblings in simplex and multiplex families throughout the United States. In both the crude and adjusted statistical models where familial clustering was statistically controlled, odds of having non-febrile seizures were significantly higher among children with idiopathic ASD than their unaffected siblings (cOR 3.48; 95% CI: 1.23 – 9.87 and aOR 5.27; 95% CI: 1.51 – 18.53). In our sample, having a history of febrile seizures increased the odds of reporting non-febrile seizures five-fold. Also, odds of non-febrile seizures increased with age, and presence of GI dysfunctions while gender was not significantly associated with the prevalence of non-febrile seizures.

Multiple studies have reported high prevalence rates of seizures in children with ASD [12, 32–35]. However, prior studies assessing prevalence of seizures in ASD populations either did not differentiate between febrile and non-febrile seizures or control for history of febrile seizures [36], or did not use unaffected siblings as the reference in their estimates [12, 37]. Lastly, in prior studies, children affected with ASD secondary to such neurological disorders as cerebral palsy, Landau-Kleffner, tuberous sclerosis, or fragile X syndrome, all of which present a higher risk of seizures independent of ASD, were included with children with idiopathic ASD, thus overestimating the reported prevalence [12, 36–39]. To address these limitations, we used unaffected siblings from the same family as a reference group, at the same time excluding those with non-idiopathic ASD. We also controlled for the confounding effects of history of febrile seizures, age, presence of GI dysfunctions, and gender in our analyses. The use of unaffected siblings as a comparison group allowed us to determine whether the observed higher prevalence of seizures in children with idiopathic ASD is a general familial genetic trait or a condition specific to the ASD phenotype. The five-fold odds of having non-febrile seizures among children with idiopathic ASD compared to their unaffected siblings suggests that the outcome is predominantly ASD-specific. However, a higher prevalence of seizures among unaffected siblings than observed in the general population (~1%), indicates some familial aggregation of the seizure phenotype [40]. More research is needed to better understand which specific risk factors contribute to these findings. Research into the possible influences of metabolic abnormalities, dysfunctions in immune system, or gastrointestinal and sleep disorders, on the risk of developing non-febrile seizures in children with idiopathic ASD is imperative as some of these risk factors may be treatable. Early detection and amelioration of risk factors has the potential to prevent seizure onset while improving long-term health outcomes among children with ASD.

### Strengths and limitations

#### Limitations

Though AGRE participants were recruited nationwide, the sample is not representative of the US population as most of the families were recruited in regions where Autism Speaks has significant activity. The data also came from largely multiplex families, limiting reported findings to families with more than one child affected with ASD. Our secondary analyses performed with data from multiplex families alone were consistent with our primary results and, though point estimates were slightly higher, the exclusion of simplex families did not change the results appreciably. In addition, some of the data collection was based on parental report rather than direct assessment and not all medical histories were verified. The use of secondary data poses certain limitations. Classification of seizure type was limited by the way the questions were asked which varied between the children with ASD and their unaffected siblings. In our case, some of the variables needed were unavailable or had high levels of missing data. For example, prior studies identified (along with age and gender) moderate-to-severe intellectual disability, motor deficits, and severe receptive language disorder among the risk factors for seizures in the ASD population [7]. High percent of missing data or absence of specific measures for some of these conditions prevented us from including these factors into models. Lastly, our exclusion criteria for non-idiopathic ASD are limited by the criteria used by AGRE, and it is possible that some residual information bias was present.

#### Strengths

Our study used a population whose ASD diagnoses were uniformly verified with gold-standard diagnostic instruments. All children were tested by AGRE physicians trained by a research-certified ADOS/ADI-R trainer to confirm the diagnosis they received from their physician prior to inclusion into the registry. The tests were electronically scored and rerated for validity and reliability. The AGRE reliability protocol requires re-rating ten percent of the ADI-R and ADOS assessments conducted by AGRE for each audit. The assessments were scored and validated through the Internet System for Assessing Autistic Children (ISAAC), thereby eliminating manual scoring errors. In addition, the use of AGRE, one of the largest data repositories containing genetic and phenotypic data on ASD, allowed a very large sample for our analyses.

By using unaffected siblings as a reference group, we were able to produce more accurate and conservative estimates of prevalence odds ratios. This method allowed us to account for differences while controlling for shared environment and genes, since siblings share both. It also enabled us to separate phenotypical characteristics specific to ASD from the general phenotypes due to familial aggregation. Furthermore, since febrile seizures represent an independent risk factor for developing non-febrile seizures later in life [18, 19], we controlled for them when estimating the odds of having non-febrile seizures among children with ASD compared to their unaffected siblings.

The SAS PROC SURVEY methods allow for variance estimation when working with complex survey sampling that does not meet the assumption of a simple random sample of an infinite population [41]. The use of these methods allowed us to cluster families while computing between-family life-time prevalence of non-febrile seizures and adjust for the effects of non-independent observations. Treating each family as a cluster provided full statistical control of not only the main effects of measured family-level confounding factors but also the unmeasured factors such as, for instance, shared environment.

## Conclusion

Our study found a five-fold higher lifetime prevalence of non-febrile seizures in children with idiopathic ASD from largely multiplex families compared to their unaffected siblings. These findings suggest that the reported non-febrile seizures may be ASD-specific and cannot be explained by genetic predisposition alone. We also found a higher prevalence of non-febrile seizures among unaffected siblings than reported for the general population, which suggests some degree of familial aggregation of genetic and phenotypic characteristics. However, the ASD-specific effect on prevalence was much stronger than the effect of familial aggregation. This study provides clear evidence for health care professionals as well as parents of children with ASD that suggest higher vulnerability of children with idiopathic ASD to non-febrile seizures. Such evidence can inform further research aimed at elucidating risk factors for non-febrile seizures in children with idiopathic ASD and possible approaches for reducing these risk factors. Future research is needed to identify risk early in life, to possibly ameliorate risk, and to prevent the onset of seizures in the ASD population.

## Abbreviations

ADI-R: Autism Diagnostic Interview-revised
ADOS: Autism Diagnostic Observation Schedule
AGRE: Autism Genetic Resource Exchange
ASD: Autism spectrum disorder
CI: Confidence interval
CSWS: Continuous spike-and-wave syndrome
DSM-IV-TR: Diagnostic and Statistical Manual of Mental Disorders, 4th edition, Text Revised
GI: Gastrointestinal
SNRPN: Small nuclear ribonucleoprotein polypeptide N
